# Effect of Longitudinal Crack Width Variation on Bond Behavior Degradation Due to Rebar Corrosion in Reinforced Concrete

**DOI:** 10.3390/ma18184335

**Published:** 2025-09-16

**Authors:** Tomohisa Kurihara, Ryusei Mitani, Toshiyuki Kanakubo

**Affiliations:** 1 Graduate School of Systems and Information Engineering, University of Tsukuba, Ibaraki 305-8573, Japan; s2420863@u.tsukuba.ac.jp; 2 Obayashi Corporation, Tokyo 108-8502, Japan; 3 Department of Engineering Mechanics and Energy, University of Tsukuba, Ibaraki 305-8573, Japan

**Keywords:** rebar corrosion, crack width, pullout test, bond stress–slip model, bond analysis

## Abstract

Pullout bond tests using specimens with an expansion-agent-filled pipe (EAFP) simulating the cracking due to rebar corrosion were conducted to evaluate the deterioration of bond behavior when the crack width is not uniformly distributed along the longitudinal direction. The primary specimens for the pullout test are designed with a bond length equal to 20 times the bar diameter. To investigate the distribution of bond stress along the rebar in detail, a bond analysis was performed using the local bond stress–slip model as a function of the induced crack width that is developed based on the pullout test of the specimens with a bond length of four times the rebar diameter. The EAFP simulation showed a tendency for larger crack widths at the free end, likely due to filling the expansion agent from the load-end side. From the results of the pullout bond test, as the induced crack width increases, the maximum bond stress decreases. The results of the bond analysis, assuming the five patterns of crack width distributions along the longitudinal direction, showed that the bond stress–slip curve is little affected by the difference in the crack width distribution. Within a bonded length up to 20 times the rebar diameter, the differences in crack width variations had little effect on the distribution of the local bond stress. It is possible to evaluate the bond behavior based on the average crack width.

## 1. Introduction

Corrosion of steel rebar is one of the primary factors of deterioration in reinforced-concrete (RC) structures. Corrosion of rebar causes damage that shortens the service life of RC structures and poses a significant risk to structural safety. When rebars are exposed to harsh environments, expansive corrosion products are generated, which can lead to cracking of the cover concrete and a reduction in the confinement around the rebar [1,2,3]. In addition to the loss of confinement, corrosion reduces the cross-sectional area of rebars, thereby diminishing the mechanical interaction between the rebar and the concrete [4,5,6]. Furthermore, previous studies have shown that the combined effects of concrete cracking, reduction in cross-sectional area, and the formation of a rust layer cause the deterioration of bond behavior in RC members [7,8,9,10]. This degradation in bond performance compromises the ability to transfer tensile and compression forces between the rebars and the concrete, leading to a reduction in load-carrying capacity and ductility of RC members.

Over the past thirty years, research on the bond performance degradation caused by rebar corrosion has progressed with increasing rapidity. In particular, some studies have developed prediction models for bond strength degradation that show good agreements with experimental results [11,12,13]. The relevant literature in this field has been reviewed by Lin et al. [14,15]. As seen in their review, the prediction models show considerable variation depending on the researcher. In many studies, accelerated electrical corrosion techniques have been used to evaluate the degradation of bond strength with corroded rebars. Naturally, the experimental results are considered to be influenced by various factors such as those mentioned earlier, e.g., effects of concrete cracking, reduction in cross-sectional area, and formation of a rust layer. Even when focusing on concrete cracking, its occurrence can vary in many ways, and it is considered that the crack width is not uniform even within one specimen. These multiple factors that affect bond behavior make it difficult to clarify the evaluation of bond performance degradation.

In contrast, the possibility of evaluating bond deterioration based on the crack width on the concrete surface has also been proposed by the researchers. Wang et al. experimentally investigated the effect of corrosion-induced cracks on bond performance and reported that an increase in crack width leads to a reduction in bond strength [16]. Law et al. investigated the effect of crack width, comparing confined and unconfined rebars, and showed that the bond strength degradation was remarkable, especially in unconfined ones [17]. Desnerck et al. reported that not only the crack width but also the location of cracks influence the distribution of bond stress and bond strength [18]. Lin et al. proposed a method to evaluate the bond strength of corroded rebars using the surface crack width as an indicator and pointed out that the surface crack width serves as an effective predictor of bond strength [19]. Among these studies, however, none have clearly discussed the bond behavior deterioration when the crack width varies within one specimen.

**Figure 1:**
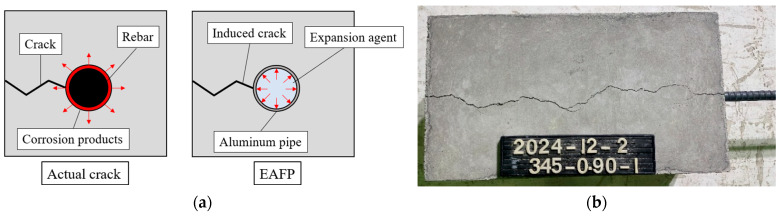
Cracking simulation by EAFP: (**a**) cracking mechanism; (**b**) example of an induced crack.

Syll et al. proposed the use of an expansion-agent-filled pipe (EAFP) as an alternative to electrochemical corrosion techniques to simulate cracking caused by corrosion [20,21]. This method involves embedding an aluminum pipe into the concrete and filling it with an expansion agent. Figure 1 shows an example of an induced crack by an EAFP. The expansion pressure generated inside the concrete simulates the volumetric expansion caused by rebar corrosion, leading to crack formation in the concrete. The advantage of the EAFP method is its ability to control the location and width of the induced cracks easily. Moreover, by using crack width as a parameter to evaluate deterioration, the effects of rust formation and accumulation can be excluded, allowing the bond behavior degradation to be assessed independently. Ultimately, Syll et al. proposed a prediction formula for bond strength degradation that reflects both the crack width and the confinement effect provided by stirrups [22].

However, even in these studies, the crack width used for evaluation was taken as the average value of the specimen, and variations along the longitudinal direction were not considered. In reality, structural members exhibit non-uniform crack widths along their length. To date, no studies have examined the effect of longitudinal crack width variation on bond behavior. In beam tests simulating induced cracks using EAFP, a distribution of crack widths was observed along the specimen length, indicating that the cracks were not uniform [23].

In this study, pullout bond tests using specimens with EAFP are conducted to evaluate the deterioration of bond behavior when the crack width induced by rebar corrosion is not uniformly distributed along the longitudinal direction. The primary specimens for the pullout test are designed with a bond length equal to 20 times the bar diameter (hereafter, called long-specimens). Furthermore, to investigate distribution of bond stress along the rebar in detail, bond analysis is performed using a sequential integration calculation based on the equilibrium condition between rebar and concrete, as well as the compatibility of slip and deformation. Since this analysis requires a local bond stress–slip relationship for cracked concrete, a preliminary pullout test is conducted using specimens with a bond length equal to four times the bar diameter (hereafter, called short-specimens).

## 2. Pullout Bond Test of Short-Specimens

To obtain the local bond stress–slip relationship corresponding to a given crack width required for the bond analysis, pullout tests using short-specimens were conducted.

### 2.1. Outline of Pullout Test of Short-Specimens

#### 2.1.1. Used Concrete and Steel Rebar for Short-Specimens

The objective of this study is to investigate the deterioration of aged concrete structures that have been in long-term service since their construction in earlier decades. The target compressive strength of concrete was set to 18 MPa, resulting in a water–cement ratio of 0.785. The mixture’s proportion of concrete is shown in Table 1. The compressive strength and splitting tensile strength were measured using cylindrical test pieces of φ100 mm × 200 mm, in accordance with JIS A 1108 [24] and JIS A 1113 [25]. The average compressive and splitting tensile strengths were 19.7 MPa and 2.13 MPa, respectively. The used rebar is a deformed bar (D16) with a nominal diameter of 15.9 mm, specified as SD345 in JIS G 3112 [26]. The mechanical characteristics of the rebar are listed in Table 2.

#### 2.1.2. Specimens, Loading, and Measurement for Short-Specimens

**Figure 2:**
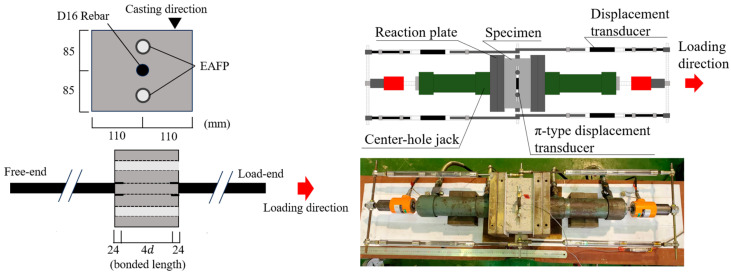
Pullout bond test (short-specimen).

Figure 2 shows the short-specimen and loading method. The cross-section is a rectangle of 170 mm × 220 mm. One deformed rebar (D16) is placed at the center of the section. The length of the specimen along the axis is 112 mm, and 24 mm of unbonded regions are set at both ends of the specimen. The length of bonded region is 64 mm, which corresponds to four times the rebar diameter. Two aluminum pipes with an outer diameter of 22 mm and a thickness of 1 mm were embedded parallel to the rebar, 50 mm from the center of the rebar, and filled with an expansion agent to induce concrete cracking. The parameter is the induced crack width, and six specimens were prepared. A Teflon sheet was placed between the specimen’s end surface and reaction plate to avoid the lateral confinement due to the reaction plate. The center-hole jack was set, and the tested rebar was clamped to the pullout load. Though Figure 2 shows the setup for the cyclic pullout loading, the one-way pullout loading in the current study was performed using only one of the center-hole jacks. The measurement items were the pullout load by a load cell, relative displacement between the specimen’s side surface and the rebar ends by displacement transducers, and the crack width on the specimen’s surface using a π-type displacement transducer. The relative displacement measured on the side opposite to the loading direction is the free-end slip. The load-end slip is obtained by adding the elongation of the rebar in the bonded region to the free-end slip assuming a uniform bond stress distribution.

**Figure 3:**
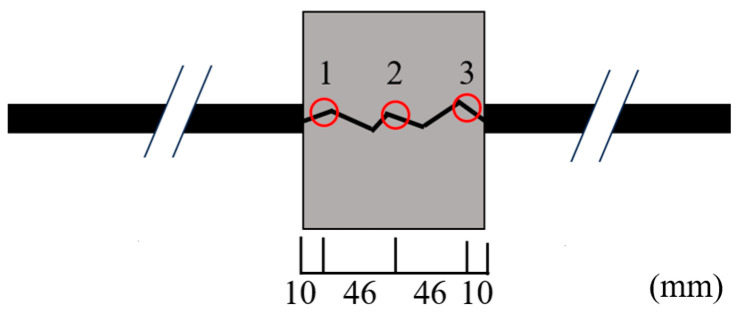
Crack width measurement points (short-specimen).

Figure 3 shows the crack width measurement points (shown by red circles) induced by EAFPs. The crack width on the specimen surface was measured using a crack scale with a precision of ±0.05 mm. The crack width was measured at three points, the central position, and 10 mm from the specimen surfaces. The crack width focused on in this study is only the surface width, and the distribution of crack widths inside the concrete is not considered. Loading was started when the average crack width reached the target value. The target values in the current test are 0 mm (no crack), 0.2 mm, 0.4 mm, 0.6 mm, 0.8 mm, and 1.0 mm.

### 2.2. Result of Pullout Test of Short-Specimen

**Figure 4:**
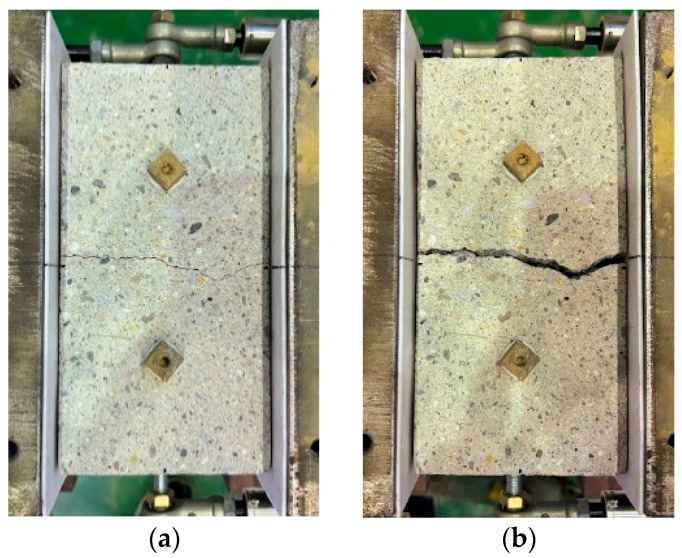
Cracks in the 0.8 mm specimen: (**a**) induced crack; (**b**) specimen after loading.

Figure 4 shows an example of the short-specimen before and after pullout loading. In all the specimens with crack induced by EAFP, the existing crack opened by pullout loading, and the pullout load reached the maximum with pulling out the rebar.

**Figure 5:**
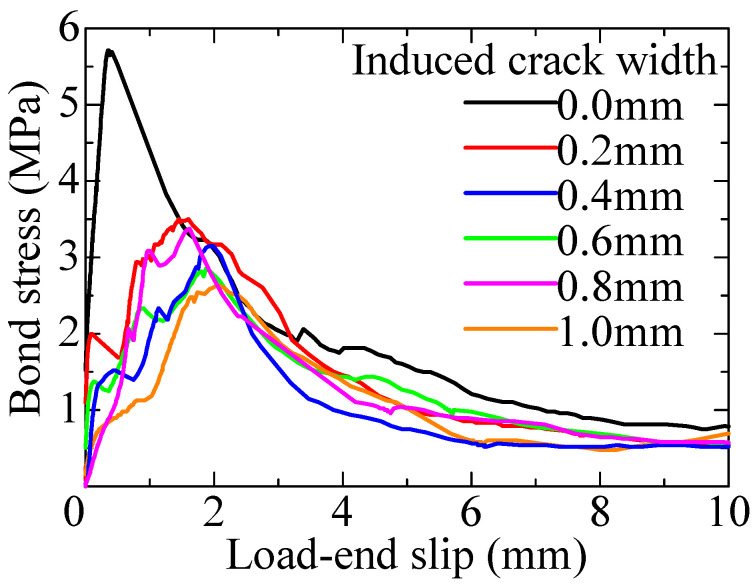
Bond stress–load-end slip curve.

Figure 5 shows the bond stress–load-end slip curves obtained from pullout test. The bond stress was calculated by dividing the pullout load by the surface area of the rebar within the bonded region. Excel files of the experimental data are provided as the Supplementary Materials. Table 3 lists the test results. The overall trend clearly demonstrated that the maximum bond stress decreased with an increasing crack width. However, in the specimens with crack widths of 0.6 mm and 0.8 mm, a slight reversal in the results was observed, which is considered to be due to variability in the crack widths and local heterogeneity of the concrete. This degradation trend is consistent with previous research findings. These bond stress–slip curves obtained from a short-specimen with a bonded length four times the rebar’s diameter are regarded as representing the local bond behavior and are used as the basis for the bond constitutive model in the bond analysis.

## 3. Pullout Bond Test of Long-Specimens

Pullout tests were conducted using long-specimens with a distributed crack width along the rebar axis.

### 3.1. Outline of Pullout Test of Long-Specimens

#### 3.1.1. Used Concrete and Steel Rebar for Long-Specimens

The long-specimens were fabricated the same way as the short-specimens. The concrete mixture proportion was also the same as that used for the short-specimens. The average compressive and splitting tensile strengths were 17.1 MPa and 1.95 MPa, respectively.

The test parameters for the long-specimens included the yield strength of the tested rebars. The used rebars are deformed bars (D16) with a nominal diameter of 15.9 mm, specified as SD295 and SD345 in JIS G 3112 [26]. The mechanical characteristics of the rebar are listed in Table 4.

#### 3.1.2. Specimens, Loading, and Measurement for Long-Specimens

**Figure 6:**
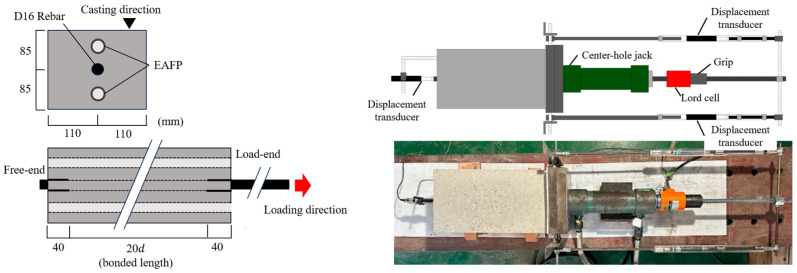
Pullout bond test (long-specimen).

Figure 6 shows a long-specimen and loading method. The cross-section is the same as that of the short-specimens, with dimensions of 170 mm × 220 mm. One deformed rebar (D16) was placed at the center of the section. The length of the specimen along the axis is 400 mm, and 40 mm of unbonded region was set at both ends of the specimen. The length of the bonded region is 320 mm, which corresponds to 20 times the rebar diameter. The cross-sectional dimensions of the aluminum pipes and the use of an EAFP are the same as those of the short-specimens. The parameters are the yield strength of the rebars and induced crack width. A total of 24 specimens were prepared, with two specimens for each parameter. The loading and measurement method are the same as those used for the short-specimens, except for the measurement method of the free-end slip. The displacement transducer for measuring the free-end slip was directly attached to the end on the free-end side of the specimen, as shown in Figure 6.

**Figure 7:**
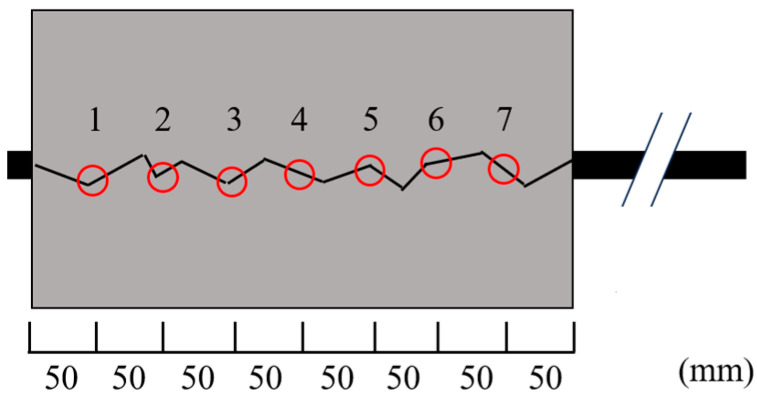
Crack width measurement points (long-specimen).

Figure 7 shows the crack width measurement points shown by red circles. The induced crack width was measured at seven positions, spaced 50 mm apart from the free-end. Loading was started when the average crack width at the seven points reached the target value. The target values in the current test are 0 mm (no crack), 0.1 mm, 0.2 mm, 0.3 mm, 0.6 mm, and 0.9 mm.

### 3.2. Results of Pullout Test of Long-Specimens

#### 3.2.1. Distribution of Induced Crack Width

**Figure 8:**
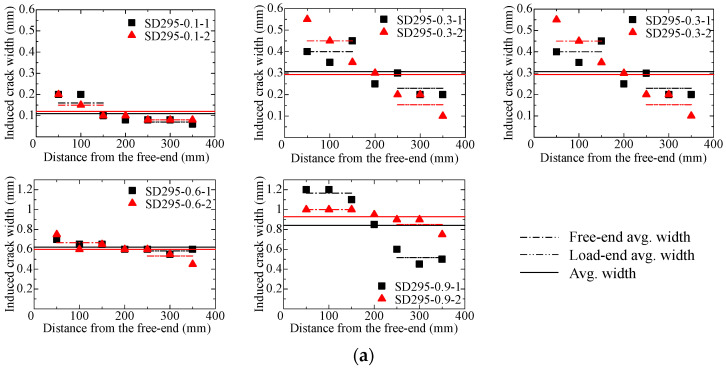

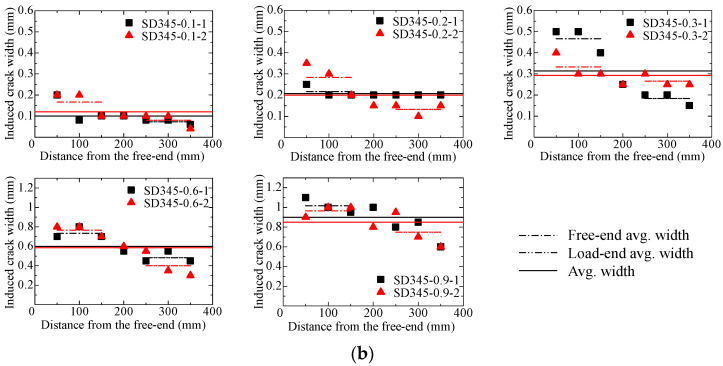
Crack width distribution in the long-specimens: (**a**) SD295 specimen; (**b**) SD345 specimen.

An example of the specimen (SD345 rebar, 0.9 mm crack width) with a crack induced by the EAFP is already shown in Figure 1a. Figure 8 shows the induced crack width distribution of all of the cracked specimens. One diagram includes the results of two specimens with same parameters. The average induced crack width at the three points on the free-end side (50 mm, 100 mm, and 150 mm from the free-end) is represented by a dash-dot line, while that on the load-end side (250 mm, 300 mm, and 350 mm from the free-end) is represented by a dash-double-dot line. A tendency for larger crack widths on the free-end side was observed, which is considered to be the effect of standing the specimen upright and filling the expansion agent from the load-end side. It is considered that the increased concentration of the agent at the lower part before the reaction causes the chemical reaction to proceed further, resulting in an increase in pressure.

#### 3.2.2. Failure Patterns and Maximum Bond Stress

The results of the pullout test are summarized in Table 5. There are two types of failure modes due to pullout loading, as follows: pullout after rebar yielding (Yield → Pullout) and pullout before rebar yielding (Pullout). Pullout loading did not cause any new cracks. In specimens with induced crack by EAFP, those cracks widened by loading. In some specimens where the rebar yielded, the rebar at the grip thinned due to the Poisson effect, making it impossible to continue gripping, which led to the termination of loading (Yield → Slip at grip). In the specimens where pullout occurred, the bond strength was fully developed, whereas in the specimens that experienced slip at the grip, the loading was terminated before the bond strength could be fully mobilized.

The average bond stress in the table is calculated by dividing the pullout load by the surface area of the rebar within the bonded region. Generally, the average bond stress at the maximum load decreased as the induced crack width increased. In the SD295 specimens, rebar yielding was observed when the crack width was 0.3 mm or less, while in the SD345 specimens, rebar yielding was observed in some cases when the crack width was 0.2 mm.

#### 3.2.3. Bond Stress–Displacement Relationship

**Figure 9:**
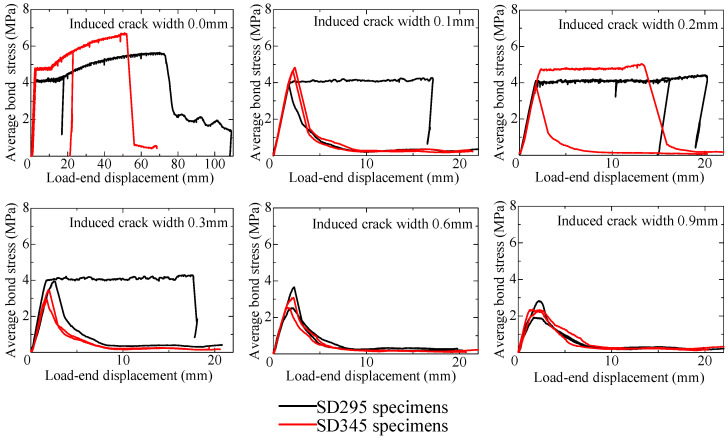
Average bond stress–load-end displacement curve of long specimens.

The average bond stress–load-end displacement curves are shown in Figure 9. The average bond stress is calculated by dividing the pullout load by the surface area of the rebar within the bonded region. The load-end displacement is the average of the displacements measured by two displacement transducers at the load-end, as shown in Figure 6, including the pullout slip and elongation of the rebar. Excel files of the experimental data are provided as the Supplementary Materials.

For specimens with induced cracks widths of 0.3 mm or less, both pullout specimens and yielding specimens are observed. It can be seen, generally, that as the induced crack width increases, the maximum average bond stress decreases. For specimens where rebar yielding occurs, the curve shows a plateau and significantly indicates differences in rebar yield strength. On the other hand, for specimens where pullout occurs before yielding, the curve shows minimal influence from differences in rebar yield strength and does not exhibit a consistent trend.

## 4. Bond Analysis Considering Crack Width Distribution

As explained in the long-specimen pullout test in the previous section, the target crack width of the specimen was set as the average value of the crack widths. However, in reality, there is variation along the longitudinal direction, as shown in Figure 8. In this section, the influence of the local distribution of crack widths on the average bond behavior is discussed through bond analysis using sequential integration. The bond stress–slip curves obtained from the short-specimen pullout test are modeled to express the local bond constitutive laws, and bond analysis considering crack width distribution is conducted using this model.

### 4.1. Modeling of Local Bond Stress–Slip Relationship

**Figure 10:**
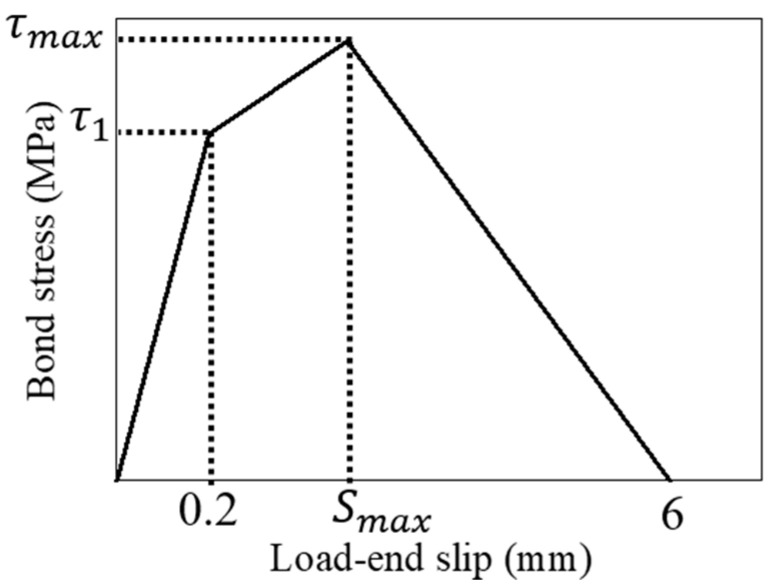
Trilinear model for local bond stress–slip curve.

The bond stress–slip relationship model is based on the experiment results from short-specimens and is represented by a trilinear model consisting of three straight lines determined by the three characteristic points, as shown in Figure 10. For all specimens with different induced crack widths, a decrease in stiffness was observed up to a load-end slip of 0.2 mm. Therefore, $\tau_{1}$ is defined as the bond stress at a load-end slip of 0.2 mm. $\tau_{max}$ represents the maximum bond stress, and $S_{max}$ is the load-end slip at the maximum bond stress. After the maximum bond stress, the softening branch is determined so that the bond stress becomes 0 MPa at a load-end slip of 6 mm, as fits with the test results shown in Figure 5.

**Figure 11:**
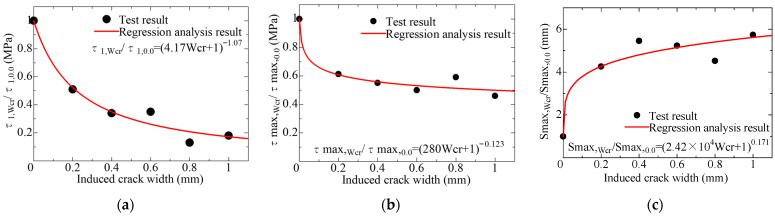
Relationships between the characteristic values and induced crack width: (**a**) $\tau_{1}$; (**b**) $\tau_{max}$; (**c**) $S_{max}$.

**Figure 12:**
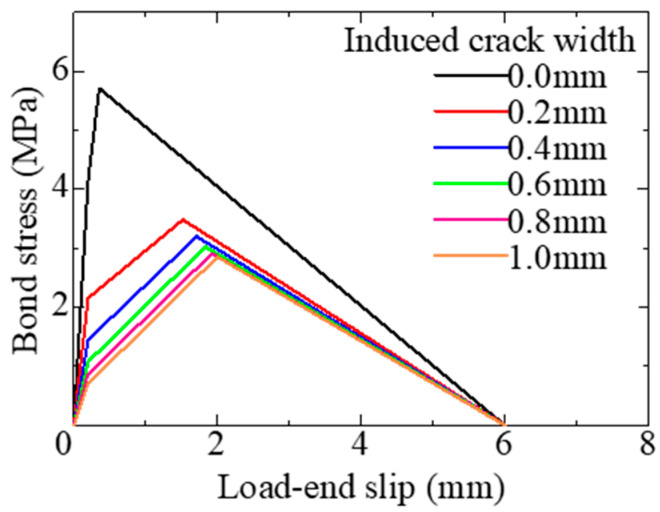
Local bond stress–slip model.

Based on the test results of the short-specimens, it is considered that these characteristic values of the model can be correlated with the crack width. Figure 11 shows the relationships between induced crack width and $\tau_{1}$, $\tau_{max}$, and $S_{max}$. The values on the y-axis are normalized by dividing them by the corresponding test results of the specimen without cracks (Wcr = 0.0). From the figures, it can be seen that both $\tau_{1}$ and $\tau_{max}$ decrease as the crack width increases, whereas $S_{max}$ increases with an increasing crack width. The results of the regression analysis for these relationships are shown in the figures. Table 6 lists the values of the characteristic points of the local bond stress–slip model by the results of the regression analysis. Figure 12 compares the local bond stress–slip model. They reflect the test results well and effectively capture the variation in shape due to the increase in induced crack width.

### 4.2. Method of Bond Analysis

This study’s analysis is conducted using a sequential integration calculation based on the equilibrium condition between the rebar and concrete, as well as the compatibility of the slip and deformation, as described in a previous study [27]. The analysis procedure for pullout test specimens is outlined as follows.

The free end of the specimen is designated as the origin, and in this analysis, an infinitesimal element, ∆x = 1 mm, is considered. A slip ($S_{0}$) is applied at the free end, and the bond stress is determined using the bond stress–slip model in that infinitesimal element. As the boundary condition, the tensile force at the free end ($P_{0}$) is zero. Next, assuming a bond stress ($\tau_{bi}$) with a corresponding induced crack width for each infinitesimal element, the tensile force ($P_{i}$) and slip ($S_{i}$) in the adjacent infinitesimal segment are determined using Equations (1) and (2), ignoring the longitudinal deformation of concrete.

(1) $$P_{i}=P_{i-1}+\tau_{bi}\cdot ∆x\cdot \varphi$$

(2) $$S_{i}=S_{i-1}+\left(\frac{P_{i-1}}{E_{s}A_{s}}+\frac{{∆P}_{i-1}}{{2E}_{s}A_{s}}\right)∆x$$

where φ is the perimeter of the rebar, $E_{s}$ is the elastic modulus of the rebar, $A_{s}$ is the cross-sectional area of the rebar, and ${∆P}_{i-1}$ is the increment between $P_{i}$ and $P_{i-1}$.

The above calculation is repeated sequentially from the free end to determine the tensile force and slip at the load end. By increasing linearly the free-end slip in small increments, it is possible to continuously obtain the tensile force and slip at the load end. The average bond stress in the analysis is also obtained by dividing the tensile force at the load end by the surface area of the rebar within the bonded region.

### 4.3. Comparison of Test Results and Analytical Results

**Figure 13:**
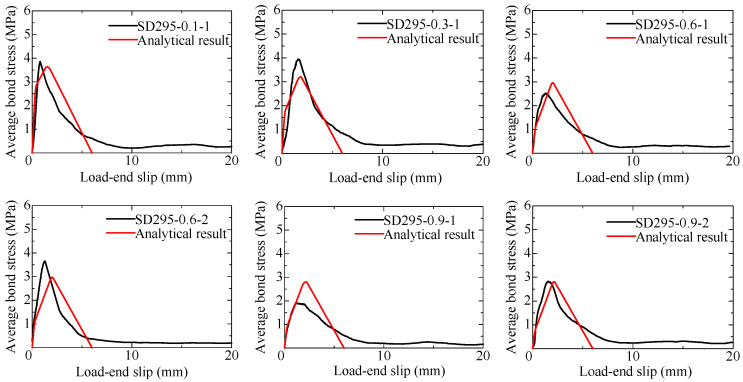
Comparison of the bond stress–slip curve of the long-specimens (SD295).

**Figure 14:**
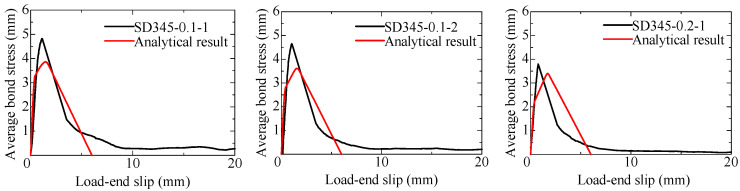

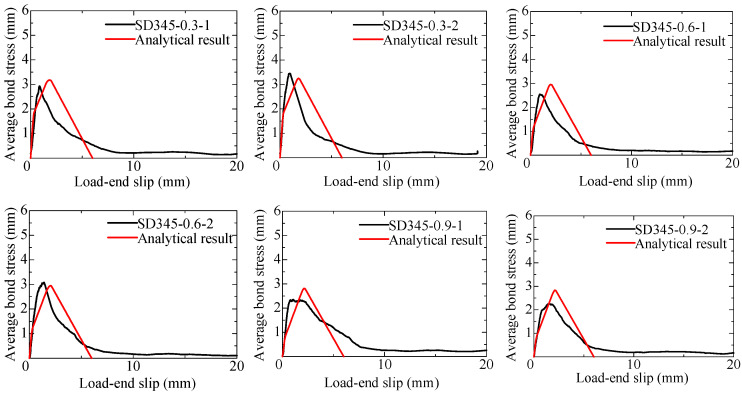
Comparison of the bond stress–slip curve of the long-specimens (SD345).

The analysis is carried out focusing on specimens where pullout occurred before yielding of the rebar. Figure 13 and Figure 14 compare the bond stress–slip curves between test results and analytical ones for the SD295 long-specimens and SD345 long-specimens, respectively. The load-end slip of the test result is obtained by subtracting the elongation of the reinforcing bar outside the bonded region from the load-end displacement. Overall, the analytical results show good agreement with the test results in terms of the maximum bond stress and the shape of the curves. Thus, it is considered that by using a local bond stress–slip model as a function of the induced crack width, the results of the specimens with a long bond length can be accurately reproduced.

### 4.4. Effect of the Distribution of the Induced Crack Width

To investigate the effect of the distribution of the induced crack width along the longitudinal direction, a bond analysis is conducted assuming the following five crack width distributions:

1. Actually observed distribution, as shown in Figure 8 (Observed);
2. Distribution to be reversed between the load end and free end (Reversed);
3. Uniform distribution of the average crack width observed (Average);
4. Uniform distribution of the maximum crack width observed (Maximum);
5. Uniform distribution of the minimum crack width observed (Minimum).

**Figure 15:**
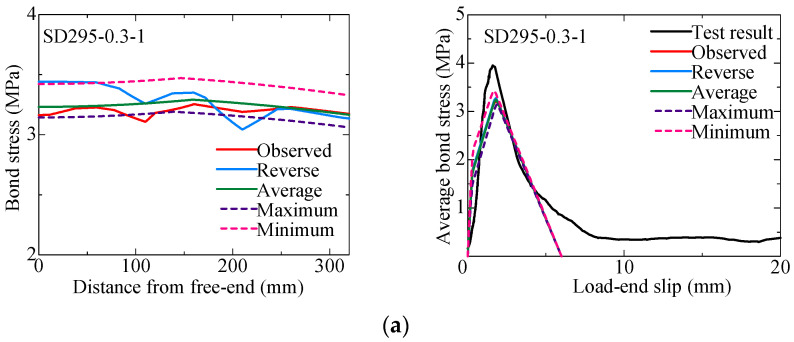

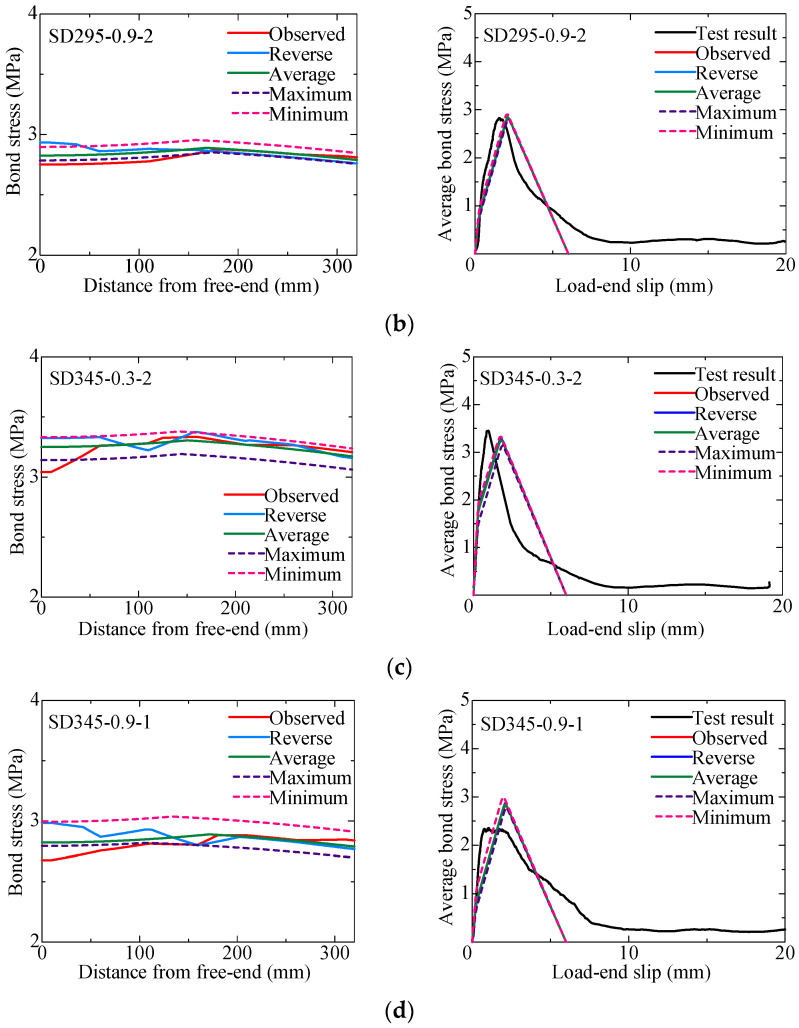
Analysis results for various crack width distributions: (**a**) SD295-0.3-1; (**b**) SD295-0.9-2; (**c**) SD345-0.3-2; (**d**) SD345-0.9-1.

Figure 15 shows the analysis results of the SD295 and SD345 specimens with induced crack widths of 0.3 mm and 0.9 mm as typical examples. The figures in the left show the analytical results of the bond stress distribution at the maximum tensile load, while the figures in the right compare the analytical and experimental results of the bond stress–slip curves.

By comparing “Observed” and “Reversed”, it can be seen that the bond stress–slip curve is little affected by the difference of crack width distribution. This result also supports that the analytical results assuming a uniform distribution with the average crack width are almost same as those of “Observed”. As a natural outcome of the analysis, the maximum bond stress is the smallest when the maximum crack width is used, and the largest when the minimum crack width is used. The analysis results of the bond stress distribution indicate that within a bonded length up to 20 times the rebar diameter, the differences in the crack width variations have little effect on the distribution of the local bond stress. As a result, it is suggested that using the average crack width over that range is sufficient for estimating the bond strength.

## 5. Conclusions

Pullout bond tests using specimens with an EAFP were conducted to evaluate the deterioration of the bond behavior when the crack width induced by rebar corrosion are not uniformly distributed along the longitudinal direction. To investigate the distribution of the bond stress along the rebar in detail, bond analysis was carried out assuming several distributions of the induced crack widths. The following conclusions are drawn based on the results of this study:

1. As the results of simulating an induced crack by the EAFP for the pullout bond specimens with bond lengths 20 times the rebar diameter, a tendency for larger crack widths on the free-end side was observed due to filling the expansion agent from the load-end side.
2. As the induced crack width increases, the maximum bond stress decreases. For specimens where pullout occurs before yielding, the bond stress–displacement curve shows minimal influence from differences in rebar yield strengths.
3. Local bond stress–slip model as a function of the induced crack width was developed based on the pullout test of the specimens with bond lengths four times the rebar diameter. The results of the specimens with a long bond length can be accurately reproduced by bond analysis using the model.
4. The results of the bond analysis, assuming the five patterns of crack width distribution along the longitudinal direction, showed that the bond stress–slip curve is little affected by the difference in crack width distribution. Within a bonded length up to 20 times the rebar diameter, the differences in crack width variations had little effect on the distribution of the local bond stress. It is possible to evaluate the bond behavior based on the average crack width.

## 6. Future Recommendations

To extend the applicability of the present findings, the influence of scaling effects should be further investigated. The current study was limited to specimens with a bonded length of 20 times the rebar diameter, within which the bond behavior could be reasonably represented by the average crack width. However, in real structural members, longer bonded lengths (e.g., 30~40 times) and more irregular crack distributions are likely to occur. Analytical modeling is, therefore, required to examine how bond stress–slip behavior may change under such conditions. This future work will help to generalize the present conclusions to larger-scale reinforced-concrete members where crack patterns are more variable.

## Figures and Tables

**Table 1:** Mixture proportion of concrete.

Water– Cement Ratio	Unit Weight (kg/m^3^)				
	Cement	Water	Fine Aggregate	Coarse Aggregate	Water Reducing Agent
0.785	248	195	930	840	2.48

**Table 2:** Mechanical characteristics of used rebar (short-specimen).

D16	Yield Strength (MPa)	Tensile Strength (MPa)	Elastic Modulus (GPa)
SD345	403	554	191

**Table 3:** Results of the pullout test of the short-specimen.

Induced Crack Width	At Maximum Load		Failure Mode
	Bond Stress (MPa)	Load-End Slip (mm)	
0.0 mm	5.71	0.355	Pullout
0.2 mm	3.50	1.525	Pullout
0.4 mm	3.15	1.953	Pullout
0.6 mm	2.85	1.874	Pullout
0.8 mm	3.38	1.619	Pullout
1.0 mm	2.63	2.054	Pullout

**Table 4:** Mechanical characteristics of used rebar (long-specimens).

D16	Yield Strength (MPa)	Tensile Strength (MPa)	Elastic Modulus (GPa)
SD295	333	468	189
SD345	378	553	186

**Table 5:** Results of the pullout test of the long-specimens.

Specimen ID		Average Bond Stress (MPa)		Failure Mode
		At Yielding	At Maximum Load	
SD295-0.0	-1 -2	4.19 4.29	5.64 4.38	Yield → Pullout Yield → Slip at grip
SD295-0.1	-1 -2	- 4.26	3.86 4.26	Pullout Yield → Slip at grip
SD295-0.2	-1 -2	4.19 4.13	4.43 4.26	Yield → Slip at grip Yield → Slip at grip
SD295-0.3	-1 -2	- 4.05	3.95 4.29	Pullout Yield → Slip at grip
SD295-0.6	-1 -2	- -	2.51 3.65	Pullout Pullout
SD295-0.9	-1 -2	- -	1.92 2.83	Pullout Pullout
SD345-0.0	-1 -2	4.79 4.78	6.71 5.72	Yield → Pullout Yield → Slip at grip
SD345-0.1	-1 -2	- -	4.82 4.64	Pullout Pullout
SD345-0.2	-1 -2	- 4.81	3.80 5.03	Pullout Yield → Pullout
SD345-0.3	-1 -2	- -	2.93 3.45	Pullout Pullout
SD345-0.6	-1 -2	- -	2.54 3.08	Pullout Pullout
SD345-0.9	-1 -2	- -	2.35 2.25	Pullout Pullout

**Table 6:** Characteristic point of the local bond stress–slip model.

Induced Crack Width *W_cr_* (mm)	$\tau_{1,Wcr}/\tau_{1,0.0}$		$\tau_{max,Wcr}/\tau_{max,0.0}$		$S_{max,Wcr}/S_{max,0.0}$	
	Test	Calc.	Test	Calc.	Test	Calc.
0.0	1.00	1.00	1.00	1.00	1.00	1.00
0.2	0.51	0.52	0.61	0.61	4.30	4.28
0.4	0.34	0.35	0.55	0.56	5.50	4.82
0.6	0.35	0.26	0.50	0.53	5.28	5.16
0.8	0.13	0.21	0.59	0.51	4.56	5.42
1.0	0.18	0.17	0.46	0.50	5.79	5.63

## Data Availability

The original contributions presented in this study are included in the article. Further inquiries can be directed to the corresponding author.

## Supplementary Materials

Excel files including the experimental data both for short specimens and long specimens can be downloaded at: https://www.mdpi.com/article/10.3390/ma18184335/s1.

## Abbreviations

The following abbreviations are used in this manuscript:

EAFP	Expansion-agent-filled pipe
RC	Reinforced concrete
